# Rate of breast biopsy referrals in female BRCA mutation carriers aged 50 years or more: a retrospective comparative study and matched analysis

**DOI:** 10.1007/s10549-021-06498-9

**Published:** 2022-04-07

**Authors:** Adi Pomerantz, Daliah Tsoref, Ahuva Grubstein, Sonya Wadhawker, Yael Rapson, Itay Gadiel, Hadar Goldvaser, Ilan Feldhamer, Ariel Hammerman, Tzipora Shochat, Eran Sharon, Inbal Kedar, Rinat Yerushalmi

**Affiliations:** 1 Institute of Oncology, Davidoff Cancer Center, Rabin Medical Center, Beilinson Hospital, Petah Tikva, 4941492 Israel; 2 Sackler Faculty of Medicine, Tel Aviv University, Tel Aviv, 69978 Israel; 3 Imaging Department, Rabin Medical Center, Beilinson Hospital, Petah Tikva, 4941492 Israel; 4 Surgery Department, Breast Cancer Unit, Rabin Medical Center, Beilinson Hospital, Petah Tikva, 4941492 Israel; 5 Chief Physician’s Office, Clalit Health Services Headquarters, Tel Aviv, 6340412 Israel; 6 Statistical Consulting Unit, Rabin Medical Center, Beilinson Hospital, Petah Tikva, 4941492 Israel; 7 Raphael Recanati Genetic Institute, Rabin Medical Center, Beilinson Hospital, Petah Tikva, 4941492 Israel

**Keywords:** Biopsy, BRCA, Breast cancer, Screening, High risk

## Abstract

**Purpose:**

To evaluate the total biopsy and positive biopsy rates in women at high risk of breast cancer compared to the general population.

**Methods:**

The study group consisted of 330 women with pathogenic variants (PVs) in *BRCA1/2* attending the dedicated multidisciplinary breast cancer clinic of a tertiary medical center in Israel. Clinical, genetic, and biopsy data were retrieved from the central healthcare database and the medical files. Patients aged 50 years or older during follow-up were matched 1:10 to women in the general population referred for routine breast cancer screening at the same age, as recommended by international guidelines. The groups were compared for rate of biopsy studies performed and percentage of positive biopsy results. Matched analysis was performed to correct for confounders.

**Results:**

The total biopsy rate per 1000 follow-up years was 61.7 in the study group and 22.7 in the control group (*p* < 0.001). The corresponding positive biopsy rates per 1000 follow-up years were 26.4 and 2.0 (*p* < 0.001), and the positive biopsy percentages, 42.9% and 8.7% (*p* < 0.0001).

**Conclusion:**

Women aged 50 + years with PVs in *BRCA1/2* attending a dedicated clinic have a 2.7 times higher biopsy rate per 1000 follow-up years, a 13.2 times higher positive biopsy rate per 1000 follow-up years, and a 4.9 times higher positive biopsy percentage than same-aged women in the general population.

## Introduction

### Background

Breast cancer is the most frequent diagnosed cancer and the leading cause of cancer-related death in women. It accounts for 23% of total cancer cases and 14% of cancer mortality worldwide [1]. In addition to clinical parameters, namely female sex, older age, and exposure to estrogen, genetic factors play an important role in breast cancer risk [2]. In the mid-1990s, genomic sequencing techniques revealed a link between germline PVs in the tumor-suppressor genes *BRCA1* and *BRCA2* with breast and ovarian cancer [3, 4]. Women with PVs in *BRCA1/2* were found to have up to an 80% lifetime chance of developing cancer, primarily of breast and ovarian origin. These PVs were inherited in an autosomal dominant manner with high penetrance.

Since then, great efforts have been invested to increase public awareness of breast cancer and to identify women at high risk, including the establishment of dedicated preventive breast clinics. Women with a family history of multiple malignancies, especially breast and ovarian carcinoma, are offered genetic counseling, and in some cases, testing is triggered by the identification of a family member with PVs in *BRCA1/2.* When a woman is identified as a carrier, she is made aware of the significance of the results, namely, the risk of developing various malignancies, the potential preventive treatment options available, and recommended follow-up algorithms. She is encouraged to start rigorous multidisciplinary follow-up from age 25 years, as recommended by international guidelines [5–7]. In our BRCA Clinic and others in Israel, this includes biannual breast examination, in addition to annual mammography, breast ultrasound, and breast magnetic resonance imaging (MRI) [8]. By contrast, in the general population, mammography screening is recommended every 2 years between the ages of 50 and 74 years [9]. Imaging findings are categorized using the Breast Imaging-Reporting and Data System (BI-RADS), with scores ranging from 0 (incomplete) to 6 [10].

The confirmation of a carrier state may be overwhelming to the patient and, indeed, constitutes a life-changing event. These women have many imaging tests during their lifetime and may well be prone to undergo an increased number of biopsies for suspicious lesions, perhaps because knowledge of the greater risk of breast cancer in these patients lowers the threshold for recommending biopsies compared to the general population. This assumption is in line with studies showing that biopsy rates are higher, and the diagnostic yield lower, in women who undergo screening MRI, regardless of a personal history of breast cancer, than in women screened with mammography alone [11]. The increase in needle biopsy rates is associated with rapidly diminishing returns in cancer detection and a marked increase in benign results. The harm caused by screening in terms of false-positive recall rates and non-cancer biopsies is true also for incident screens, but the rates are much lower [12].

### Study objective

The objectives of this study were to evaluate the rate and yield of biopsies in all women with PVs in *BRCA1/2* attending our dedicated BRCA Clinic and to compare these findings in the subpopulation of women aged 50 or more years during follow-up with same-aged women in the general population. Given their higher lifetime risk of breast cancer, we wanted to explore if women age > 50 with PVs in BRCA are more likely to undergo breast biopsy compared to average risk women in a screening program at the biggest HMO (health medical organization) in Israel.

## Methods

### Design and setting

A retrospective study was conducted at the tertiary BRCA Clinic of Davidoff Cancer Center, Rabin Medical Center in Israel which provides comprehensive follow-up, including psychological counseling, to women with PVs in *BRCA1/2,* based on international guidelines. Rabin Medical Center belongs to Clalit Health Services, the largest of four health maintenance organizations in Israel, which manages a central computerized healthcare warehouse that integrates data from its hospitals, community clinics, laboratories, and pharmacies nationwide. For purposes of this study, clinical, imaging, biopsy, and operative data were retrieved from the central database and hospital medical records.

The cohort for the first part of the study consisted of 330 consecutive women at high risk of development of breast cancer attending the BRCA Clinic between May 2000 and September 2019. Women in whom breast/ovarian cancer developed before they were identified as having a PV in *BRCA1/2* were excluded as they did not undergo follow-up at the clinic. The rate of biopsies performed (number of biopsies/screening episodes in years), including the leading test to biopsy, was calculated and the results assessed.

The second part of the study was restricted to women among this cohort who were older than 50 years during follow-up between January 2002 and June 2019. This cutoff was chosen because the screening program for women at average risk for breast cancer starts at age 50 years [5–7]. Of the 113 women who met this criterion, we excluded 34 who had reached their 50th birthday before or at the beginning of the follow-up period to ensure that all patients analyzed had undergone strict follow-up as recommended. Also excluded were 7 women who were ineligible for regular screening tests through Clalit Health Services and 11 women in whom breast cancer developed before the beginning of follow-up or before age 50 years, for a final group of 61 women.

**Fig. 1:**
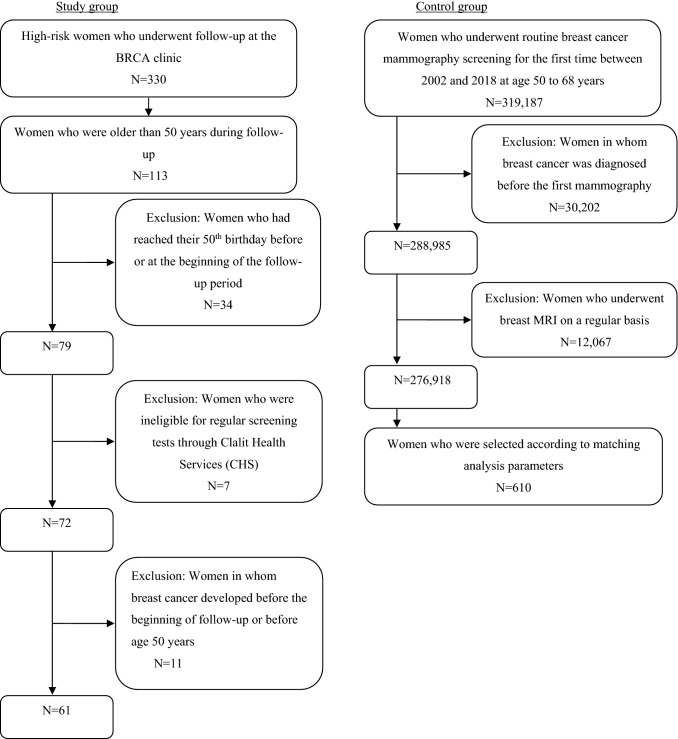
Flowchart illustrating the inclusion and exclusion criteria in each group

To form the control group for the second part of the study, we identified 319,187 women who underwent routine breast cancer mammography screening for the first time between 2002 and 2018 at age 50 to 68 years, according to the Clalit Health Services database and were followed for the same period, from January 2002 to June 2019. We excluded 30,202 women in whom breast cancer was diagnosed before the first mammography and 12,067 women who underwent breast MRI on a regular basis and were considered high risk. The inclusion and exclusion criteria for each group are presented in Fig. 1.

The 61 women aged 50 + at follow-up were analyzed for breast biopsy rate and positive biopsy rate, and the findings were compared to 610 subjects from the control group matched 1:10 for clinical characteristics with the study patients.

The study was approved by the local Institutional Ethics Committee.

The selection process for the study is shown in Fig. 1.

### Statistical analysis

The rate of biopsies performed per exposure period was compared between groups using the conditional test with mid-P adjustment. The positive biopsy ratio was calculated using chi-square test (with df = 1). Matching analysis was performed for the following parameters: age group (50, 51–55, 56–60, and 61–65 years), socioeconomic status, Charlson Comorbidity Index score (0, 1, 2–3), sector (Orthodox Jewish, non-Orthodox Jewish), use of hormone replacement therapy, and prior use of oral contraceptive pills. Sector was chosen as a parameter because orthodox Jewish women have a unique lifestyle which is suspected to impact their life time risk to develop breast cancer [13]. Statistical analyses were performed with Microsoft Excel, SPSS version 25, and WinPepi software.

## Results

### Clinical characteristics

The 330 patients in the study cohort included 198 women with PVs in *BRCA1* and 108 women with PVs in *BRCA2*; 2 women had PVs in both *BRCA1* and *BRCA2.* Of the remainder, one woman had PVs in *PTEN*, 16 had a family history of breast cancer but no recognized PVs, and 5 had a family history of PVs in *BRCA1/2* but had opted not to have a genetic test themselves. The median duration of follow-up was 5.4 years (range, 0.03–18.2 years), and the median age at the beginning of follow-up at the clinic was 37.4 years (range, 18.3–73.7 years). Table 1 shows the specific PVs identified and cancer histories of the study group.

**Table 1:** PVs identified in *BRCA1/2* and cancer history of study group (*N* = 330). *SCC* squamous cell carcinoma, *BCC* basal cell carcinoma

Characteristics	No. of patients
Mutation	
BRCA1	198
185delAG	142
5382insC	33
Tyr978X	6
Other	10
Unknown	7
BRCA2	108
6174delT	101
Other	6
Unknown	1
BRCA1 + BRCA2	2
PTEN	1
Not a carrier	16
No BRCA test	5
History of malignancy	
None	296
Ovarian cancer	7
SCC, BCC	9
Melanoma	3
Other	15

### Biopsy analysis

A total of 142 lesions were identified in the study group: 76 (53.5%) by MRI, 31 (21.8%) by physical examination, 17 (12%) by ultrasound, and 11 (7.7%) by mammography; in 7 patients (4.9%), the modality was unknown (Table 2). Of the 31 lesions discovered by physical examination, 13 were found on the first or second visit at the clinic, before MRI was performed, and 10 (32.2%) were identified a short time after a negative MRI. Of the remainder, 2 were found on self-examination, and 6 were breast skin lesions.

**Table 2:** Screening modalities leading to biopsy for 142 lesions identified. *MRI* magnetic resonance imaging

Modality	Malignant		Benign		Total	
	*N*	% of malignant	*N*	% of benign	*N*	% of total
Physical examination	7	18.9	24	22.9	31	21.8
Ultrasound	4	10.8	13	12.4	17	12
MRI	19	51.4	57	54.3	76	53.5
Mammography	6	16.2	5	4.8	11	7.7
Unknown	1	2.7	6	5.7	7	4.9

All 142 lesions were biopsied: 117 Tru-cut breast biopsies, 13 fine-needle aspirations, 8 breast skin biopsies, and 4 diagnostic lumpectomies. The median patient age at biopsy was 41.5 years. The biopsy characteristics and pathology results are presented in Table 3. A malignancy was identified in 37 samples (26.1%), including 7 prompted by physical examination. The total biopsy rate per 1000 follow-up years was 69.8. The positive biopsy rate per 1000 follow-up years was 18.2, and the positive biopsy percentage [(number of positive biopsies/number of total biopsies) × 100] was 26.1%.

**Table 3:** Biopsy characteristics in patients with *PVs in BRCA1/2. IDC* invasive ductal carcinoma, *DCIS* ductal carcinoma in situ, *ILC* invasive lobular carcinoma

Characteristics	No. (%)
Type of biopsy (*n* = 142)	
Tru-cut breast biopsy	117 (82.4)
Fine needle aspiration	13 (9.2)
Breast skin biopsy	8 (5.6)
Excisional biopsy	4 (2.8)
Type of malignancy (*n* = 37)	
Invasive	33 (89.2)
DCIS	3 (8.1)
Other	1 (2.7)
Type of benign lesions (*n* = 105)	
High-risk lesions (ADH, ALH, and LCIS)	2 (1.9)
Fibroadenoma	11 (10.5)
Others	92 (87.6)

### Comparison with the general population

Findings for the 61 women aged 50 years or more with PVs in *BRCA1/2* and 610 women of the same age who underwent routine breast cancer screening are presented in Tables 4 and 5, and the parameters used in the matched analysis are shown in Table 6. The incidence rate ratio (IRR) and significance were calculated using mid-P values. All IRRs for negative, positive, and total number of biopsies were found to be significant. The total biopsy rate per 1000 follow-up years was 61.7 in the study group and 22.7 in the control group (*p* < 0.001). The positive biopsy rate per 1000 follow-up years was 26.4 in the study group and 2.0 in the control group (*p* < 0.001), and the corresponding positive biopsy percentages [(number of positive biopsies/number of total biopsies) × 100] were 42.9% and 8.7% (*p* < 0.0001).

**Table 4:** Results of biopsy studies in the BRCA and the general population groups

Biopsy studies	Study group (*N* = 61) Years of follow-up = 340.6		Control group (*N* = 610) Years of follow-up = 6088.2	
	No	% of biopsies performed in the study group	No	% of biopsies performed in the control group
Total biopsies	21.0		138.0	
Positive biopsies	9.0	42.9	12.0	8.7
Negative biopsies	12.0	57.1	126.0	91.3

**Table 5:** Biopsy rate per 1000 years of follow-up

Biopsy studies	Study group	Control group	Ratio	*p* value
Total biopsies	61.7	22.7	2.7	< 0.001
Positive biopsies	26.4	2.0	13.4	< 0.001
Negative biopsies	35.2	20.7	1.7	0.048

**Table 6:** Parameters for matched analysis

Parameter	Study group		Control group	
	*N*	% of study group	*N*	% of control group
Age group (year)				
50	37	60.7	370	60.7
51–55	14	23.0	140	23.0
56–60	8	13.1	80	13.1
61–65	2	3.3	20	3.3
Charlson score				
0	39	63.9	390	63.9
1	13	21.3	130	21.3
2–3	9	14.8	90	14.8
Sector				
Orthodox Jewish	2	3.3	20	3.3
Non-Orthodox Jewish	59	96.7	590	96.7
HRT and OCP				
HRT	17	27.9	170	27.9
OCP	6	9.8	60	9.8

## Discussion

BRCA mutation carriers undergo intensive breast follow-up, including radiological studies such as mammography and MRI. Each test has its own false-positive rate (FPR). Mutation carriers are referred for an annual screening protocol which is associated with a higher FPR than standard screening [14, 15]. The present study evaluated the total biopsy rate, positive biopsy rate, and positive biopsy percentage among women with PVs in *BRCA1/2* compared to the general population of women referred for routine screening. We sought to investigate the biopsy load of women with PVs in *BRCA1/2* who attend a dedicated clinic.

Our matched analysis revealed that in women with PVs in *BRCA1/2*, the biopsy rate per 1000 follow-up years was 2.7 times higher than in the general population. Additionally, their positive biopsy rate per 1000 follow-up years was 13.2 times higher, and their positive biopsy percentage, 4.9 times higher. Nevertheless, it is noteworthy that despite the higher percentage of positive biopsies in the *BRCA1/2* carriers, most of the biopsies performed in this patient group at the clinic (73.9%) yielded benign results. This finding is in accordance with the 72% rate of benign biopsies reported in a study of healthy women with PVs in *BRCA1/2* followed at another high-risk clinic in Israel [16]. Others examined biopsies of women with dense breast tissue who underwent breast MRI as part of their follow-up. Although the results cannot be directly compared with ours because of the different screening structure, as in our study, the cohort was comprised of high-risk women who required MRI analysis as part of their screening program. The rates of malignant results were comparable: 26.1% in our study and 31% in the dense breast study [17]. Together, these findings reflect the need for personalized screening regimens [18]. Our review of the literature failed to reveal a matched analysis similar to that performed here.

An unexpected incidental finding of the present study was the important role of physical examination, second after MRI, in the identification of suspicious lesions during follow-up. Of note, in one-third of cases in which suspicious lesions were first discovered by physical examination, the requested MRI test was not performed on time. This occurred mainly in patients who were new to the clinic.

This study has several limitations. Besides the retrospective design, which has inherent biases due to unknown or unrecorded confounders, the main limitation of the study was our inability to perform a matched analysis for the under-50 age group because routine screening mammography in Israel is recommended only for women above this age, whereas screening in women with PVs in *BRCA1/2* starts much earlier. Furthermore, a different screening protocol was used in the two groups. The high-risk protocol includes more frequent imaging, including MRI, which is known to be associated with a relatively high prevalence of false-positive results [19]. In addition, as noted above, most of the women with PVs in *BRCA1/2* started screening at an earlier age, and the false-positive in the first year of screening is known to be higher than in later years. This may suggest that the difference between the groups is even larger than observed in this study [20]. Finally, we did not stratify the patients by duration of use of oral contraceptives and hormone replacement therapy.

The high biopsy rates found here highlight the limitations of current imaging studies, including MRI. The literature suggests that the innovations in artificial intelligence and radiomics will improve image analysis in the future, making it easier to distinguish benign from malignant small breast masses and lead to changes in the guidelines [21]. The results also imply that studies of personalized screening programs should include women with PVs in *BRCA1/2* [22].

It should be emphasized that this study was intended to mirror a given situation in the context of adherence to the national and international guidelines. From that perspective, it clearly describes the differences in biopsy load and outcomes between patients with PVs in *BRCA1/2* and the general population of women aged 50 years or more.

### Clinical implications

To our knowledge, this is the first study to evaluate total biopsy and positive biopsy rates among women aged 50 years or more with PVs in *BRCA1/*2 and without a personal history of breast cancer compared to the general population. Our data are relevant both to patients and caregivers, improving their understanding of the “carrier journey” in a dedicated clinic. Repeated imaging and biopsies are very stressful, and for some women, may have a considerable negative impact on quality of life. Providing these patients with detailed information on the process and outcomes of follow-up can contribute to their feeling of health safety and can be a major psychological predictor when considering a prophylactic surgery [23, 24]. This information is also helpful for health authorities in terms of regulating resources and costs.

The findings of the present study are particularly timely given the increasing awareness of the importance of *BRCA* testing in the high-risk population and the concerns raised regarding the use of biopsies as a screening test for women of Ashkenazi Jewish ancestry [25]. About 2.5% of Ashkenazi Jewish women have PVs in *BRCA1/2* [4], so we may expect many more such patients in the future.

The high positive biopsy percentage in our study group is in line with current international recommendations for follow-up of women with PVs in *BRCA1/2* and should be part of the quality assurance considerations of teams assessing the requirements for biopsy tests.

The study may support earlier reports suggesting that dedicated BRCA clinics effectively meet the specific short- and long-term needs of this high-risk population [8].

## Data Availability

The datasets generated during and/or analyzed during the current study are not publicly available but are available from the corresponding author on reasonable request.

## Code availability

NA.
